# Perceived effects of the COVID-19 pandemic on clinical psychology internships in Sweden

**DOI:** 10.1186/s12909-023-04236-x

**Published:** 2023-04-17

**Authors:** Hillevi Bergvall, Cornelia Larsson, Elinor Eskilsson Strålin, Benjamin Bohman, Sven Alfonsson

**Affiliations:** Centre for Psychiatry Research, Department of Clinical Neuroscience, Karolinska Institutet, & Stockholm Health Care Services, Region Stockholm, Stockholm, Sweden

**Keywords:** Psychologist, Internship, Trainee, Supervision, Pandemic, COVID-19

## Abstract

**Background:**

The COVID-19 pandemic has had an unprecedented impact on societies and health care services worldwide, including the clinical training of psychology interns. Some of the pandemic-related restrictions were in breach of the internship requirements, increasing the risk of failed internships and a shortage of new health care professionals. This situation needed to be assessed.

**Methods:**

Web-based surveys were administered to clinical psychology interns in Sweden 2020 (*n* = 267) and 2021 (*n* = 340), as well as to supervisors in 2020 (*n* = 240). The supervisors also provided information about their interns (*n* = 297).

**Results:**

Risk factors for a prolonged internship, such as pandemic-related absence from work (12.4% in 2020 and 7.9% in 2021), unqualified work (0% in 2020, 3% in 2021), and change in internship content were low. However, remote interactions using digital services increased. Face-to-face patient contacts decreased significantly from 2020 to 2021 (*Χ*^2^ = 5.17, *p* = .023), while remote work and remote supervision increased significantly (*Χ*^2^ = 53.86, *p* < .001 and *Χ*^2^ = 8.88, *p* = .003, respectively). Still, the content in patient contacts and supervision was maintained. Most interns reported no difficulties with remote supervision or supervision in personal protective equipment. However, of the interns who reported difficulties, role-play and skills training in remote supervision were perceived as significantly harder (*Χ*^2^ = 28.67, *p* < .001) than in supervision using personal protective equipment.

**Conclusions:**

The present study indicates that clinical training of psychology interns in Sweden could proceed despite a societal crisis. Results suggest that the psychology internship was flexible in the sense that it could be realized in combined face-to-face and remote formats without losing much of its value. However, the results also suggest that some skills may be harder to train in remote supervision.

**Supplementary Information:**

The online version contains supplementary material available at 10.1186/s12909-023-04236-x.

## Background

Clinical internship is an essential part of the training of psychologists, but during the COVID-19 pandemic, it may have been disrupted and challenged. The pandemic has had a yet incalculable impact on societies worldwide, affecting the lives of billions of people. The health care sector in particular was hit on several fronts [1]. To meet the emergency of the pandemic and follow the recommendations of the World Health Organization [2], the health care systems were forced to rapidly change; resources had to be relocated to manage this new disease, and measures were taken to prevent the spread. Mental health services were affected in many ways and went through significant shifts in the administration of services [3]. The need for social distancing urged a digitalization of patient contacts, staff meetings, clinical training, and supervision. For example, some early studies reported that psychiatric outpatient care switched to telepsychiatry quite rapidly [4–6]. In the US, many psychologists conducted all therapy consultations online. For example, in a US study, psychologists providing mental health services conducted 86% of their clinical work using remote modalities during the pandemic compared to 7% before the pandemic [7], and in another US study in a pediatric setting [8], psychologists shifted from delivering services 100% face-to-face before the pandemic to 82% telemedicine during the pandemic.

Studies have now started to emerge about how these changes have affected the provision of psychological treatment. For example, a scoping review of 77 studies conducted in mental health services primarily in the US and the UK concluded that remote care (mainly using video and phone calls) was perceived as highly acceptable by most clinicians and patients and that the quality of therapeutic relationships was generally good [9]. Similarly, the review reported high levels of adoption of remote care and no adverse effects on attendance rates after the introduction of telepsychiatry. Furthermore, most studies reported good feasibility and there was evidence of widespread implementation and integration of telepsychiatry into mental health services [9]. However, in some studies, there were concerns about the appropriateness of remote care (e.g., managing medication, and engaging and assessing new patients). In terms of outcomes, the review concluded that telepsychiatry could be as effective as face-to-face care in the short term, although it was noted that most studies were small [9]. Several studies in the review concerned psychologists. For example, a study conducted in Portugal [10] showed that 58% of psychologists continued to provide services to their patients during a lockdown and 76% reported the outcomes to be at least the same as those of face-to-face care. In this study, 70% of psychologists considered their experiences with remote care as positive and 30% as neither negative nor positive; none reported their experiences as negative.

Studies on remote clinical supervision during the pandemic are lacking. Generally, it has been suggested that remote supervision can adhere to existing supervision models and there is evidence to suggest that remote supervision is effective and usable in cognitive-behavioral therapy (CBT), particularly for anxiety and depression [11]. Thus, while there are several studies on the impact of the pandemic on the provision of psychological treatment, there is a lack of studies on the effects of remote supervision on psychologists. Furthermore, studies from Sweden are lacking. The Swedish context may be particularly worthy of study due to relatively milder pandemic restrictions and a society with a well-developed information technology infrastructure.

Compared to many other countries, Sweden took less restrictive measures in response to the pandemic, especially in 2020, and there was no complete lockdown. However, in line with the World Health Organization guidelines [2], the Public Health Agency of Sweden recommended keeping a social distance, working from home, and staying at home if showing any COVID-19 symptoms. Swedish health care services went through major changes in response to the pandemic. In mental health care, there was a shift to more remote contacts (i.e., via telephone or video sessions), activities such as group psychoeducation and group treatment were postponed, and some clinics restricted their intake of new referrals. Throughout Sweden, though, there was a wide variety in how recommendations were interpreted and applied. In addition, the response to the pandemic also differed, following the spread of the disease through various parts of the country resulting in different conditions for clinical practice. Meanwhile, to meet future demands, the training of new health care practitioners, such as psychologists needed to continue.

### Psychologist education and internship in Sweden

The Swedish training program in psychology is running on a full-time basis for five years (300 European Credit Transfer and Accumulation System credits) including courses in scientific methodology, cognition, human development, neuropsychology, pedagogy, social psychology, organizational psychology, clinical psychology, psychotherapy, at least 15 weeks of practice, and delivering psychological treatment to clients under close supervision. After receiving their MSc degree, all novice psychologists are required to do a one-year-long internship before they can apply for a psychologist license.

The internship aims to promote professional development and clinical skills, and the internship positions are created by routine services which employ the interns and provide an individual supervisor. The supervisor (who has at least three years of working experience as a licensed psychologist) offers support and guidance, while continuously assessing the intern’s progress toward professional independence. Because interns often have additional educational needs (e.g., how to conduct a neuropsychiatric assessment, or treatment of certain diagnoses), and supervisors struggle to keep themselves updated with the Swedish National Board of Health and Welfare’s (SNBHW) regulations, a director of studies have been appointed in each of the Swedish health care regions to run educational programs for interns, as well as support supervisors and employers.

After completion of the internship, the supervisor certifies whether the intern is fit to work independently as a psychologist. Thereafter, the intern can apply for a psychologist license with the SNBHW, which regulates and issues licenses for all clinical professions in health care services. SNBHW requires complete attendance and weekly face-to-face supervision during the internship. At least half of the internship must be allocated to clinical practice (i.e., psychological assessment and treatment), and at least a fourth of the internship must be allocated to development work (e.g., organizational work, psychological research, teaching, supervising other staff, prevention, or evaluation). Remote patient contact is accepted only as a limited part of the clinical training. All departures from these requirements are to be compensated by prolonging the internship until the requirements are met. Because prolongation of internships is applied, the SNBHW normally rejects very few license applications from psychologists educated in Sweden (e.g., 2018: *n* = 3, 0.54%; 2019: *n* = 6, 1.15%) as reported by SNBHW (e-mail B Norrbacka, July 5, 2021).

Consequently, the SNBHW’s requirements conflicted with the measures taken to prevent the spread of COVID-19. The recommendation to stay at home with any symptoms would likely increase the rate of sick leave for both interns and their supervisors, interfering with the requirements of complete attendance and weekly supervision. Large-scale non-attendance of patients raised concern that the clinical practice of interns would be insufficient, specifically regarding activities that did not easily transfer to online meetings (e.g., assessment of cognitive functions or group treatment). Initially, the SNBHW signaled that the requirements for internships would remain unchanged during the pandemic. Further on, the SNBHW communicated a less restrictive and more flexible attitude to evaluate applications for approval of internships, regarding pandemic-related adjustments in healthcare. However, it was unclear which requirements would be affected and how, as SNBHW never gives advance notice regarding approvals. Thus, the path towards a psychologist license seemed uncertain, when the quality and completion of the internship were at risk by measures taken to prevent the spread of COVID-19, a concern that received attention in the Swedish Network of Directors of Studies for the Psychology Internships, as well as causing worry among interns and their supervisors.

### Aims

The primary aim of the present study was to explore how psychology interns and their supervisors perceived that the COVID-19 pandemic affected their ability to comply with the authorities’ requirements regarding attendance, continuation, supervision, and content of the internship, and ultimately the risk of not meeting the requirements for licensure. The secondary aims were to explore how the pandemic affected the interns’ perceived workload, work tasks, and supervision content.

## Methods

### Study design, setting, and procedure

A cross-sectional study of all psychology internships in all 21 Swedish regions was conducted using online surveys. A first survey was administered from 17 August to 4 October 2020 after the first wave of the COVID-19 pandemic had ebbed away. As most internships start in autumn, only the last part of those internships was affected by the pandemic. We also wanted to assess how the pandemic affected the internships conducted mainly during the pandemic (i.e., interns starting after the first survey was conducted). Therefore, a second survey to a different set of interns was administered from 30 April to 4 June 2021, as the third wave was slowing down in most parts of Sweden. In addition, a survey targeting supervisors of the interns was administered from 22 October to 9 December 2020 while the second wave of the pandemic was slowly rising in Sweden. Responding to the surveys was voluntary and anonymous.

The Swedish Network of Directors of Studies for the Psychology Internships is a forum for all regional representatives to discuss internships and regulations, continuously as well as during a twice-yearly conference. During the pandemic, the directors discussed pandemic-related concerns, both present and anticipated. To assess these concerns, the authors and directors of studies HB and CL created the items making up the surveys in this study. The directors were informed of the surveys and invited to provide feedback on the survey items during the design phase. Overall, the feedback was positive but led to a few minor adjustments in item wording and an added response alternative to two items. No psychometric testing or piloting of the surveys was performed, as an overview of the interns’ situation in Sweden was needed swiftly to manage the situation. An e-mail including study information and a survey link was then sent to the directors, who distributed the e-mail to all their interns and supervisors. Six interns responded to the survey on paper and their responses were entered manually into the online survey platform.

The Network of Directors of Studies and the SNBHW have regular contact to enable dialogue, cooperation, and quick distribution of information. However, some employers (usually in the private health care sector) have no director of studies, who could inform their interns about the survey. That is unless a regional director of studies informed those interns because they participated in a regional program (depending on its relevance, cost, and ability to invite additional participants).

### Participants

In 2020, 267 psychology interns responded to the first survey, representing almost half of approximately 600 interns or 15 of the 21 (71.4%) Swedish regions. In 2021, 340 interns responded to the second survey, representing 56.7% of the interns or 20 (95.2%) Swedish regions. The respondents had completed on average two-thirds of their 12-month internship (in 2020: *M* = 8.9 months; in 2021: *M* = 8.6 months). A total of 45 interns were practicing during both surveys for interns, allowing for a potential overlap of respondents in the two groups. A majority were employed by the regional health care services and practiced their internship in psychiatric care.

A total of 240 supervisors responded to the survey in 2020, representing 14 (66.7%) Swedish regions, also providing information on the 297 interns they had supervised during the pandemic. Supervisors were licensed psychologists with at least three years of clinical work experience. They had supervised a median of one intern (interquartile range [IQR] = 0, max = 4) during the pandemic and two interns (IQR = 4, max = 20) so far during their careers. Given the similar response patterns of interns and supervisors in 2020, we chose to target only interns in 2021. For information on internship settings, as reported by interns and supervisors respectively, see Table 1.

**Table 1:** Work Fields, Patient/Client Age Groups, Employers, and Swedish Regions Among Interns and Supervisors

Internship Setting	Interns 2020		Interns 2021		Supervisors 2020	
	*n*	%	*n*	%	*n*	%
Work field						
Psychiatric care	165	62.0	190	55.9	197	66.3
Primary care	60	22.6	92	27.1	60	22.6
Habilitation	19	7.1	33	9.7	20	6.7
Rehabilitation	11	4.1	12	3.5	12	4.0
Somatic health care	9	3.4	15	4.4	9	3.0
Research and education	9	3.4	8	2.4	4	1.3
School psychology	7	2.6	13	3.8	5	1.7
Social services	0	0.0	7	2.1	1	0.3
Patient/client age group						
Children (< 18 years)	126	47.4	177	52.1	142	47.8
Adults (18–65 years)	171	64.3	225	66.2	188	63.3
Older adults (≥ 65 years)	29	10.9	61	17.9	46	15.5
Employer						
Regional health care	212	79.7	278	81.8	248	83.5
Private health care, public funding^1^	37	13.9	41	12.1	38	12.8
Private health care, private funding	8	3.0	4	1.2	5	1.7
Community/Schools	7	2.6	12	3.5	5	1.7
State/governmental	4	1.5	5	1.5	3	1.0
Swedish region						
Main capital region (Stockholm)	100	37.6	98	28.8	144	48.5
Western region (Gothenburg)	60	22.4	54	15.9	0	0.0
Other regions^2^	106	39.8	186	54.7	153	51.5

^1^ Private health care services in Sweden can be contracted to provide tax-funded public health care.

^2^ Thirteen regions in 2020 for both interns and supervisors, and 18 regions in 2021, each region representing up to 8.4% of the respondents.

### Assessments

The 2020 survey for interns consisted of 15 items regarding their work situation, see Supplementary for more details. We used multiple-choice items for information on their internship period and setting (region, employer, work field, and patient/client age group). We asked about the number of days affected in terms of absence, remote work, unqualified work, and furloughs.

Additional items included how the pandemic affected the content of the internship (1 = *no or minimal impact*, 2 = *content is affected but the internship as a whole complies with SNBHW’s requirements*, 3 = *clinical work is at risk of being less than 50%*, 4 = *development work is at risk of being less than 25%*, *and* 5 = *clinical work is at risk of being less than 50%, and development work being less than 25%*; single choice question), specific work tasks regarding assessment, treatment, development work tasks (1 = *not at all affected* to 5 = *completely affected*), the workload (1 = *much lower* to 5 = *much higher*), frequency of remote contact with patients or supervisor (1 = *almost never* to 5 = *almost always*), frequency of supervision being cancelled (1 = *almost never* to 5 = *almost always*), frequency of sessions drifting in focus towards the pandemic and its effects with patients or supervisor (1 = *never* to 5 = *almost always*), and the extent of excessive focus drift with patients (1 = *almost all focus was on COVID-19* to 5 = *focus remained on presenting complaint*).

We also collected data on the content of supervision sessions before the pandemic (retrospectively reported) and during the pandemic regarding support, validation, encouragement, relevant feedback, prioritizing and planning, professional guidance, development of skills matching work during the internship, methods of assessment, treatment, and development work, and whether the supervision was perceived as rewarding (1 = *not at all* to 5 = *very much*). Finally, we also collected data on the availability of managers, supervisors, responsible psychologists or psychiatrists, and directors of studies.

The 2021 survey targeting interns consisted of 17 items, largely the same as in the survey for interns in 2020. A starting point (year and month) of the internship was added, whereas items on the content of pre-pandemic supervision and furlough were no longer applicable and thus removed. New items were added, since the Swedish authorities in 2021 recommended all health care staff to work in personal protective equipment (PPE; i.e., face mask and/or visor): how often PPE was used with patients or the supervisor (1 = *almost never* to 5 = *almost always)*, how PPE affected concentration and the working relationship with patients (1 = *not at all* to *harder* 5 = *very much harder*), and how PPE and remote supervision affected the working relationship with the supervisor, the understanding of instructions or feedback, performing role-play and other types of skills training (1 = *not at all harder* to 5 = *very much harder*).

The survey targeting supervisors in 2020 consisted of 21 items and included largely the same items as the survey for interns in 2020, only rephrased for supervisors. However, supervisors had to answer both for themselves and for every intern they supervised regarding the work field, patient/client age group, employer, and region for the supervisor and intern(s), respectively, as well as the number of supervised interns during the pandemic and their entire career. Further, supervisors were asked to which extent they had to ensure that intern(s) could maintain qualified tasks, had to protect the intern(s) from too much work and responsibility, needed to consult a director of studies on pandemic-related questions regarding the internship or supervision, and had more/less time for the intern(s) (1 = *strongly disagree* to 5 = *strongly agree*). Data on the number of days with supervisors’ absence, remote work, and remote supervision because of the pandemic was collected. Finally, we also collected data regarding supervisors’ belief that the supervision was rewarding for the interns, if the interns had stated the supervision was rewarding, and whether the conditions for the supervision had changed before and after the pandemic (1 = *not at all* to 5 = *very much*). The supervisor had to answer all questions one time per supervised intern. Finally, the number of failed internships from 2018 to 2021 was collected from the SNBHW (e-mail B Norrbacka, July 5, 2021).

### Statistical analysis

Descriptive statistics were calculated for all variables. Since not all data were normally distributed, analysis was conducted using non-parametric tests. Chi-square tests were used to analyze group differences in proportions on categorical variables. Mann-Whitney tests were used to analyze group differences on continuous and ordinal variables. A *p*-value of < 0.05 was used as a threshold for statistical significance in all analyses and effect sizes for Mann-Whitney tests were calculated with *r* = *z*/√*N*, where *z* is the standardized test statistic and *N* is the total sample size on which *z* is based. *r*-values of 0.10, 0.30, and 0.50 were interpreted as small, medium, and large, respectively. To correct for multiple comparisons, the false discovery rate was investigated by comparing the *p*-values with the Benjamini-Hochberg Adjusted *p*-values and the results were only reported as significant if they did not reach the calculated threshold value [12]. The SPSS (Version 26, SPSS Inc., Chicago, IL) was used for all statistical analyses.

## Results

### Risk factors for not complying with the internship requirements

Pandemic-related absence from work for more than 10 days was reported by 12.4% of psychology interns in 2020 and 7.9% in 2021, a decrease over time that was not statistically significant. Unqualified work was almost non-existent for interns in both 2020 and 2021 (0% and 0.3%, respectively) and none were furloughed. Most interns maintained seeing most of their patients face-to-face in 2020 (91.6%) but this proportion decreased significantly (*Χ*^2^ = 5.17, *p* = .023) to 84.4% in 2021. Consistent with these results, the proportion of interns who performed remote work (e.g., from home) for more than 10 days increased significantly (*Χ*^2^ = 53.86, *p* < .001) between 2020 (4.1%) and 2021 (26.5%). Overall, weekly supervision continued throughout the pandemic, with only a small number of interns experiencing frequent pandemic-related cancellations in 2020 (3.8%) and 2021 (1.5%). Remote supervision increased significantly (*Χ*^2^ = 8.88, *p* = .003) from 7.9% to 2020 to 15.9% in 2021. See Table 2 for frequencies of perceived risk factors, failures, and other pandemic-related effects on internships from the perspective of interns and supervisors, respectively.

**Table 2:** Pandemic-Related Effects on Internships from the Perspectives of Interns and Supervisors

Variable	Interns 2020		Interns 2021		Supervisors 2020	
	*n*	%	*n*	%	*n*	%
Internships at risk						
Absence > 10 days	33	12.4	27	7.9	29	9.8
Unqualified work^1^ > 10 days	0	0.0	1	0.3	0	0.0
Remote work > 10 days	11	4.1	90	26.5	16	5.4
Supervision frequently canceled	10	3.8	5	1.5	8	2.7
Frequent^2^ remote supervision	21	7.9	54	15.9	16	5.4
Internships failing to meet requirements						
Prolongation > 10 days	20	7.5	24	7.1	20	6.7
Requirements not met	15	5.6	17	5.0	9	3.0
Internships affected regarding work and supervision						
Increased workload	40	15.0	63	18.6	24	8.1
Decreased workload	74	27.8	77	22.6	59	19.9
A large effect^3^ on psychological assessments	12	4.5	13	3.8	3	1.0
A large effect^3^ on psychological treatments	21	7.9	22	6.5	6	2.0
A large effect^3^ on development work^4^ tasks	26	9.8	29	8.5	10	3.4
Frequent^2^ patient focus drift	0	0	6	1.8	0	0
Frequent^2^ supervision focus drift	5	1.9	5	1.5	1	0.3
At least of half patient contacts in PPE^5^	-	-	203	59.7	-	-
Supervision frequently in PPE^5^	-	-	123	36.2	-	-

^1^Unqualified work = assisting inpatient care or acute somatic care.

^2^Frequent = responses of “4. *At least half*” to “5. *Almost always*”.

^3^A large effect = responses of “4. *Very affected*” to “5. *Completely affected*”.

^4^Development work = organizational work, research, teaching, supervising staff, prevention, or evaluation.

^5^PPE = Personal protective equipment.

### Failure to meet requirements and prolongation of the internship

While 5.6% and 5.0% of the interns in 2020 and 2021, respectively, reported that their internship did not meet the SNBHW’s requirements, the supervisors reported that 3.0% of the internships in 2020 did not. This did not vary significantly between work fields, patient/client age groups, employers, or regions. However, the pandemic caused a prolongation of some internships (7.5% in 2020 and 7.1% in 2021). The SNBHW reported that only one intern (0.17%) was denied a psychologist license in 2020 and none in 2021.

### Effects on work and supervision

See Table 2 for pandemic effects on work and supervision. More than half of the interns reported that the pandemic did not affect their workload. For the remaining interns, the workload was either increased or decreased. With few exceptions, the pandemic only had a small effect on work tasks during 2020 and 2021, generally perceived as a change in psychological treatments and development work. Interns maintained treatment focus in most of their patient contacts, with only a few interns in 2021 experiencing an excessive focus on the pandemic in most of their patient work.

Most supervision maintained its intended focus, such as professional development and psychological work, and very few experienced an excessive pandemic-related focus shift.

In 2021, PPE was used in most face-to-face patient contacts by most interns (59.7%), as it had been introduced to governmental recommendations by that time. In 2021, over a third of interns used PPE in supervision, however, 50.0% of the interns never did.

The supervisory conditions changed during the pandemic, according to half of the supervisors (*n* = 131, 54.6%). Specifically, some supervisors experienced less time for their intern (*n* = 13, 5.4%), more time for their intern (*n* = 16, 6.7%), a need to ensure that the intern could maintain qualified work tasks (*n* = 16, 6.7%), a need to protect the intern from excessive workload (*n* = 14, 5.8%), a need to consult a director of studies in pandemic-related questions regarding the internship (*n* = 16, 6.7%), and a need to receive support in their supervisory role (*n* = 3, 1.3%). Both interns and supervisors perceived support functions to be highly available (i.e., manager, director of studies, supervisor, and senior colleagues).

Among the interns receiving occasional remote supervision (*n* = 174, 51.2%) in 2021, most perceived this to interfere only “a little” or “not at all” with understanding instructions (*n* = 164, 94.3%), understanding feedback (*n* = 162, 93.1%), building a working relationship (*n* = 151, 86.8%), and performing role-play or skills training (*n* = 102, 61.1%). Among the interns receiving supervision in PPE (*n* = 170, 50%), most perceived this to interfere only “a little” or “not at all” with understanding instructions (*n* = 160, 94.1%), understanding feedback (*n* = 158, 92.9%), building a working relationship (*n* = 147, 86.5%), and performing role-play or skills training (*n* = 145, 85.3%). The proportions of interns reporting that understanding instructions or feedback, or building a working relationship was “a lot harder” or “very much harder” in remote supervision than in supervision using PPE, were not significantly different, see frequencies in Table 3. However, the proportion of interns that found role-play and skills training to be “a lot harder” or “very much harder” was significantly larger (*Χ*^2^ = 28.67, *p* < .001) in remote supervision than in supervision in PPE.

**Table 3:** Effects of Remote Contacts and Personal Protective Equipment on Supervision According to Interns 2021

Supervisory format	Not at all harder		A little harder		Harder		A lot harder		Very much harder	
	*n*	%	*n*	%	*n*	%	*n*	%	*n*	%
Remote supervision (*n* = 174)										
Supervisory relationship	86	49.4	65	37.4	16	9.2	5	2.9	2	1.1
Understanding instructions	105	60.3	59	33.9	8	4.6	2	1.1	0	0.0
Understanding feedback	107	61.5	55	31.6	9	5.2	3	1.7	0	0.0
Role-play and skills training	63	37.7	39	23.4	33	19.8	24	14.4	8	2.4
Supervision in PPE (*n* = 170)										
Supervisory relationship	89	52.4	58	34.1	17	10.0	2	1.2	4	2.4
Understanding instructions	128	75.3	32	18.8	6	3.5	1	0.6	3	1.8
Understanding feedback	123	72.4	35	20.6	7	4.1	2	1.2	3	1.8
Role-play and skills training	103	62.0	42	25.3	16	9.6	3	1.8	2	1.2

*Note.* PPE = Personal protective equipment

Interns reported significantly higher levels of supervision support, validation, encouragement, relevant feedback, competence development, and that supervision was rewarding to interns in 2021 compared to 2020, while differences in prioritization and planning, professional guidance, assessment methods, treatment methods, and development methods remained statistically non-significant (see Table 4).

**Table 4:** Means of Supervision Content According to Interns

Variable	Interns 2020 (*n* = 261)			Interns 2021 (*n* = 340)				
	*M*	*SD*		*M*	*SD*	*z*	*p*	*r*
Support	3.91	1.07		4.15	0.95	2.76	0.006	0.16
Validation	3.75	1.12		4.06	1.02	3.26	0.001	0.19
Encouragement	3.83	1.06		4.12	0.99	3.43	0.001	0.20
Relevant feedback	3.62	1.10		3.89	1.01	2.91	0.004	0.17
Prioritization and planning	3.42	1.11		3.60	1.03	1.74	0.082	0.10
Professional guidance	3.76	1.01		3.75	1.01	0.07	0.944	0.01
Competence development	3.34	1.13		3.58	1.03	2.32	0.020	0.13
Assessment methods	3.47	1.04		3.54	1.07	0.79	0.430	0.05
Treatment methods	3.44	1.04		3.52	1.02	0.71	0.478	0.04
Development methods	2.92	1.14		2.90	1.08	0.12	0.904	0.01
Rewarding to intern	3.95	1.06		4.21	0.98	3.00	0.003	0.17

## Discussion

Overall, the results of the present study showed that the pandemic did not have any substantial negative influence on the psychology interns or their supervisors. Pandemic-related absence from work was rather low and work tasks for interns remained largely unchanged. Most interns continued to see their patients face-to-face and receive weekly supervision. A few interns and supervisors were concerned that the requirements of the internship were not met, however only one license application was denied in the period 2020–2021. Most interns did not perceive the occasional remote supervision or supervision in PPE to interfere substantially in terms of understanding instructions or feedback from supervisors, building a working relationship, or performing role-play or skills training. However, more interns perceived role-playing or skills training to be more difficult in remote supervision than face-to-face wearing PPE.

Given that some of the routines introduced during the pandemic might stay with us, it could be important to consider possible differences between the formats of supervision, and therefore not exclusively use remote supervision. However, despite being a part of psychotherapy practice for over a decade, there has been no empirical research on the efficacy of online supervision in this area [13, 14]. There is also sparse research on how psychotherapists and their supervisors view online technology for supervision [15] but there is an emerging understanding that online supervision requires a specific skill set and context compared to traditional face-to-face supervision [16]. Whether and how online supervision should be used in the post-pandemic world is still poorly understood and in need of more research.

The results of the present study were somewhat surprising, as we expected a larger perceived impact of the pandemic on psychology internships [17]. There are several possible explanations. First, Sweden had no lockdowns and most of the health care services engaging psychology interns remained open. No psychology internships were terminated, in contrast to, for example, some US states where facilities had to close in the early phase of the pandemic, thereby ceasing all clinical training [18]. Second, the Network of Directors of Studies consulted the SNBHW and took preventive action in recommending how the internship could meet the requirements despite any disturbances due to the pandemic. Third, similar to other organizations such as the US Association of Psychology Postdoctoral and Internship Centers [7], the SNBHW was less strict in its assessment of internships during the pandemic, provided that the supervisor declared that any deviations from the requirements were due to the pandemic. Fourth, Sweden is a society highly permeated by information technology, which may have facilitated the transition from face-to-face contact to remote interactions. For example, 94% of the population aged 16 to 85 years old had access to the Internet at home in 2020 and 88% used the Internet daily [19].

The rapid transition to providing psychological services using digital formats in various health care settings observed in previous studies [5, 7] was also evident among psychology interns in Sweden. Moreover, the experiences of switching to remote interactions for treatment delivery were generally positive, a finding which is consistent with other reports in various mental health provider populations and settings [9]. Considering the lack of previous studies on the effects of the pandemic on remote supervision in psychologists, the finding of positive experiences in psychology interns is important.

Future practice should learn from the positive experiences during the pandemic of the present study and consider incorporating remote formats of treatment delivery and occasional remote supervision into routine practice and standard training. Such change in practice would provide more flexibility and thus robustness in times of crisis [20], including a smooth transition to total use of remote care and supervision if needed. In addition, for some service users, for example, individuals with autism, severe anxiety disorders, physical disabilities, or geographical barriers, telepsychiatry can be preferable [9].

The present study had some limitations to be considered when interpreting the results. First, different samples of interns provided the data in 2020 and 2021; thus, we cannot know to what extent observed changes represented true changes perceived as dependent on the effects of the pandemic. Second, and relatedly, data were only collected during the pandemic, and we had no “off-pandemic” comparison data. Thus, we cannot know whether responses reflected pandemic-specific effects since they were based solely on interns’ perception of any changes in the work situation. Third, interns outside of the directors of studies oversight were not included which might have affected the results. Finally, data were self-reported and may thus be subjected to recall bias or social desirability bias. Generalizability may be limited to Swedish psychology interns and their supervisors, or other settings characterized by similar requirements for internship, health care organization, pandemic effects, and pandemic-related restrictions. At the same time, the present study had several strengths, as half of the psychology interns in Sweden responded to the survey, covering most Swedish regions, and as results showed similar response patterns in interns and supervisors, indicating valid and reliable data.

The present study provides some valuable lessons for the future, in the case of other pandemic events, and confirms some previous findings. First and foremost, psychology internships can continue if the health care services and regulatory authorities provide some flexibility regarding guidelines and requirements [21, 22]. Also, guidelines and requirements need to be updated to better reflect the future demands of health care workers. For example, experiences of remote treatment and working with PPE are valuable experiences that may prepare psychologists for working under different circumstances and to be flexible and adapt to changing demands in society at large [23, 24].

## Conclusions

Most psychology interns could continue their internships during the COVID-19 pandemic in Sweden, with only a few experiencing risk factors resulting in a prolonged internship. However, remote contact with patients and supervisors increased. A minority of the participants reported parts of remote supervision to be somewhat more difficult compared to face-to-face or PPE, such as role-playing and skills training, for reasons yet to be investigated. The present study is encouraging in the sense that it indicates that clinical training may proceed despite a societal crisis, at least in clinical training of psychology interns in Sweden. The study also suggests that the psychology internship is flexible in the sense that it can be realized in a combined face-to-face and remote format without losing much of its value. Future research may involve a direct comparison between face-to-face and digital formats of clinical practice of psychology interns and their supervision using an experimental design to further explore the potential benefits of remote interactions [25].

## Electronic supplementary material

Below is the link to the electronic supplementary material.

Supplementary Material 1

## Data Availability

The datasets analyzed during the current study are not publicly available due to issues of confidentiality but are available from the corresponding author upon reasonable request, assuming it is in accordance with applicable Swedish laws and regulations.

## Declarations

### Authors’ information

HB and CL are members of the Swedish Network of Directors of Studies for the Psychology Internships: HB is a director of studies for the psychology internships in Region Stockholm and CL is a director of studies for the psychologists of Psychiatry Southwest, Region Stockholm.

## List of abbreviations

PPE: Personal Protective Equipment
SNBHW: Swedish National Board of Health and Welfare
