# Supplementary material for: Perceived effects of the COVID-19 pandemic on clinical psychology internships in Sweden
Source: BMC Med Educ. 2023 Apr 17;23:249. doi: 10.1186/s12909-023-04236-x (PMC10106869; doi:10.1186/s12909-023-04236-x)
Supplement: Supplementary file 1 — Supplementary Material 1 [file 12909_2023_4236_MOESM1_ESM.docx]

# Appendix

## The Survey to Interns in 2020

**1. Region of your internship**

| ○ Blekinge | ○ Kronoberg | ○ Värmland |
| --- | --- | --- |
| ○ Dalarna | ○ Kalmar | ○ Västerbotten |
| ○ Gotland | ○ Norrbotten | ○ Västmanland |
| ○ Gävleborg | ○ Skåne | ○ Västra Götaland |
| ○ Halland | ○ Stockholm | ○ Örebro län |
| ○ Jämtland Härjedalen | ○ Sörmland | ○ Östergötland |
| ○ Jönköping | ○ Uppsala |  |

**2. Employer of your internship**

○ Regional
 ○ Private
 ○ Private, providing regionally funded health care

○ Community/schools
 ○ State/governmental
 ○ None of the above, please specify: ___________

**3. Your internship ends (month)**:

○ Jan ○ Feb ○ Mar ○ Apr ○ May ○ Jun ○ Jul ○ Aug ○ Sep ○ Oct ○ Nov ○ Dec

**4. Your internship ends (year)**

○ 2020 ○ 2021 ○ 2022

**5. Work field of your internship** (*multiple choice possible)*

| □ Psychiatric care | □ Habilitation | □ Somatic health care | □ School psychology |
| --- | --- | --- | --- |
| □ Primary care | □ Rehabilitation | □ Research & Education | □ Social services |
| □ Occupational health care | □ None of the above, please specify: ____________ | | |

**6. Patient or client group of your internship** (*multiple choice possible)*

| □ Children < 18 years | □ Adults 18-65 years | □ Older adults > 65 years |
| --- | --- | --- |

**7. Your absence, remote work, and furlough during the pandemic**

|  | Number of days |
| --- | --- |
| You were absent during your internship due to the pandemic | ____ |
| You conducted “unqualified work tasks” due to the pandemic   (e.g., reallocated to assisting inpatient or acute somatic care) | ____ |
| You worked remotely during your internship due to the pandemic | ____ |
| You were on furlough due to the pandemic | ____ |
| Your internship will be extended due to the pandemic | ____ |

**8. The content of your internship was affected by the pandemic**

○ No or minimal impact
○ Content is affected, but the internship complies with SNBHW’s requirements
○ Your clinical work is at risk of being less than 50%
○ Your development work is at risk of being less than 25%
○ Your clinical work is at risk of being less than 50% and your development work being less than 25 %

**9. Internship work tasks were affected by the pandemic**

Internship work tasks may be affected when patients, colleagues, or working partners are absent, thus interfering with service provision, or work projects. Work may also be affected by cancelling group activities or projects. There may also be a change in routines, patient groups, or psychologists' work tasks, or resources may be reallocated.

A response of "completely affected" means that an internship could not be carried out for a period, e.g., closure of a service unit or relocation of an intern.

|  | Not at all affected | Slightly affected | Moderately affected | Very affected | Completely affected |
| --- | --- | --- | --- | --- | --- |
| Psychological assessment work | ○ | ○ | ○ | ○ | ○ |
| Psychological treatment work | ○ | ○ | ○ | ○ | ○ |
| Development work tasks | ○ | ○ | ○ | ○ | ○ |

**10. Your workload has been affected by the pandemic**

○ Much lower (e.g., work tasks were reduced or removed)

○ Lower (e.g., cancelled appointments, cancelled meetings)
○ No effect (alternatively: periods of lower and higher workload balancing each other out)
○ Higher (e.g., more appointments, substituting for absent colleagues)
○ Much higher (e.g., more patients, added work tasks, substituting for colleagues to a large extent)

**11. Supervision and patient sessions were affected by the pandemic**

|  | Almost never | Rarely | Some-times | At least half | Almost always |
| --- | --- | --- | --- | --- | --- |
| Weekly 1h supervision was cancelled | ○ | ○ | ○ | ○ | ○ |
| Supervisory sessions over telephone/video call (instead of physical meetings) | ○ | ○ | ○ | ○ | ○ |
| Supervisory sessions focused too much on the pandemic | ○ | ○ | ○ | ○ | ○ |
| Patient sessions over telephone/video call (instead of physical meetings) | ○ | ○ | ○ | ○ | ○ |
| Patient sessions had to focus on the pandemic and/or its societal effects, instead of primary concern or treatment | ○ | ○ | ○ | ○ | ○ |

**12. Your supervision included the following BEFORE the pandemic outbreak**

|  | Not at all | A little | Sufficient | A lot | Very much |
| --- | --- | --- | --- | --- | --- |
| Supporting you | ○ | ○ | ○ | ○ | ○ |
| Validation you | ○ | ○ | ○ | ○ | ○ |
| Encouraging you | ○ | ○ | ○ | ○ | ○ |
| Relevant, concrete feedback on your work | ○ | ○ | ○ | ○ | ○ |
| Prioritization and planning of your work | ○ | ○ | ○ | ○ | ○ |
| Professional guidance as a psychologist | ○ | ○ | ○ | ○ | ○ |
| Skill development matching your work tasks | ○ | ○ | ○ | ○ | ○ |
| Methods for psychological assessment | ○ | ○ | ○ | ○ | ○ |
| Methods for psychological treatment | ○ | ○ | ○ | ○ | ○ |
| Methods for development work | ○ | ○ | ○ | ○ | ○ |
| Supervision was rewarding for you | ○ | ○ | ○ | ○ | ○ |

**13. Your supervision included the following AFTER the pandemic outbreak**

|  | Not at all | A little | Sufficient | A lot | Very much |
| --- | --- | --- | --- | --- | --- |
| Supporting you | ○ | ○ | ○ | ○ | ○ |
| Validation you | ○ | ○ | ○ | ○ | ○ |
| Encouraging you | ○ | ○ | ○ | ○ | ○ |
| Relevant, concrete feedback on your work | ○ | ○ | ○ | ○ | ○ |
| Prioritization and planning of your work | ○ | ○ | ○ | ○ | ○ |
| Professional guidance as a psychologist | ○ | ○ | ○ | ○ | ○ |
| Skill development matching your work tasks | ○ | ○ | ○ | ○ | ○ |
| Methods for psychological assessment | ○ | ○ | ○ | ○ | ○ |
| Methods for psychological treatment | ○ | ○ | ○ | ○ | ○ |
| Methods for development work | ○ | ○ | ○ | ○ | ○ |
| Supervision was rewarding for you | ○ | ○ | ○ | ○ | ○ |

**14. The content of your patient contacts was changed due to the pandemic and/or its societal impact**

○ Almost entirely focused on COVID-19

○ Focused mostly on COVID-19

○ Focused to some extent on COVID-19

○ The focus was only slightly affected by COVID-19

○ The focus remained on presenting complaint, despite COVID -19.

**15. The following people were available when needed during the pandemic (outside of scheduled appointments)**

○ Manager

○ Internship supervisor

○ Attending physician or psychologist, responsible for patients

○ Director of studies

## The Survey to Interns in 2021

The 2021 survey included 17 items, largely the same as the 2020 survey for interns. However, the following adjustments were made:

- A starting point (year and month) of the internship was added, and items no 3 and 4 were merged into one item (year and month) when the internship ended/was planned to end.
- For item 7, the subitem of “You were on furlough due to the COVID-19 pandemic” was removed since it was no longer relevant in 2021.
- Item 12 was removed, and item 13 was slightly rephrased, since almost no interns in April 2021 would have started their one-year internship before the pandemic, and retrospective comparisons could not be made. Item 13 was therefore rephrased into “Your supervision has included the following:”.
- For item 11, the following subitem was added: “Supervision was carried out in protective equipment (PPE) (facemask and/or visor)”.
- New items were added, when Swedish authorities recommended all health care staff to work in personal protective equipment in 2021 (see items 13-15 below).
- Item 15 was removed, as the supportive functions were highly present in 2020, we expected them to be even more present in 2021, thus making the item redundant.

**13. Wearing personal protective equipment (facemask and/or visor) affected your contact with patients**

|  | Not at all harder | A little harder | Harder | A lot harder | Very much harder |
| --- | --- | --- | --- | --- | --- |
| Establishing a good alliance with patients in PPE | ○ | ○ | ○ | ○ | ○ |
| Concentrating on work with patients in PEE | ○ | ○ | ○ | ○ | ○ |

**14. Supervision via telephone or video call**

|  | Not at all harder | A little harder | Harder | A lot harder | Very much harder |
| --- | --- | --- | --- | --- | --- |
| Establishing a good relationship via telephone/video | ○ | ○ | ○ | ○ | ○ |
| Understanding instructions via telephone/video | ○ | ○ | ○ | ○ | ○ |
| Understanding feedback via telephone/video | ○ | ○ | ○ | ○ | ○ |
| Conducting role-play and other skill-training via telephone/video | ○ | ○ | ○ | ○ | ○ |

**15. Supervision wearing personal protective equipment (facemask and/or visor)**

|  | Not at all harder | A little harder | Harder | A lot harder | Very much harder |
| --- | --- | --- | --- | --- | --- |
| Establishing a good relationship in PPE | ○ | ○ | ○ | ○ | ○ |
| Understanding instructions in PPE | ○ | ○ | ○ | ○ | ○ |
| Understanding feedback in PPE | ○ | ○ | ○ | ○ | ○ |
| Conducting role-play and other skill-training in PPE | ○ | ○ | ○ | ○ | ○ |

## The Survey to Supervisors in 2020

The survey targeting supervisors in 2020 consisted of 21 items. It included largely the same items as the survey for interns in 2020, rephrased for supervisors. However, a few changes were made:

- Items 1 (region), 2 (employer), 7 (absence/remote/furlough), 10 (workload), and 15 (support) were duplicated, for supervisors to respond first regarding themselves and later regarding their intern(s). A few subitems were not relevant and thus removed for supervisors: for item 7 the subitems on unqualified work tasks and prolonged internship, and for item 15 availability of supervisors and attending physician/psychologist.
- Supervisors were asked to report how many interns they had supervised during the COVID-19 pandemic and throughout their career.
- One item was added for supervisors, please see item 6 below.
- For all other items, supervisors responded regarding their intern(s).
- For items 12 and 13, the following subitems were added: “You believe the supervision was rewarding for the intern”, “Your intern reported the supervision to be rewarding” and “Your workplace conditions for supervising were good”.

**6. Supervisory conditions changed by the COVID-19 pandemic (from March 2020 onwards)**

|  | Strongly disagree | Disagree | Neutral | Agree | Strongly agree |
| --- | --- | --- | --- | --- | --- |
| You had more time for the intern due to the pandemic | ○ | ○ | ○ | ○ | ○ |
| You had less time for the intern due to the pandemic | ○ | ○ | ○ | ○ | ○ |
| You had to make an effort for the intern to maintain qualified work tasks during the pandemic | ○ | ○ | ○ | ○ | ○ |
| You needed to protect the intern from excessive workload/responsibility because of the pandemic | ○ | ○ | ○ | ○ | ○ |
| You needed to consult your director of studies on pandemic-related issues | ○ | ○ | ○ | ○ | ○ |
| You needed support as a supervisor from your director of studies due to the pandemic | ○ | ○ | ○ | ○ | ○ |
| You noticed no changes in the conditions for you as a supervisor due to the pandemic | ○ | ○ | ○ | ○ | ○ |
